# Mr. Fielding, on Hooping Cough

**Published:** 1801-02

**Authors:** Geo. Fielding

**Affiliations:** Surgeon. Hull


### *To the Editors of the Medical and Phyfical Journal.*

GENTLEMEN,

TO a great majority of your readers, a defcription of the Hooping-cough would be totally unneceffary; I beg, however, through the medium of your ufeful Journal, to offer to their confideration, what I conceive will be found a very valuable remedy for this difeafe.

My attention has been turned towards this fubject, on account of its having been twice epidemic in this town during the laft eight years; and becaufe, during thefe times, I have heard it very frequently mentioned as one of the opprobria medicorum.—I have made trial of feveral different medicines.

Emetics (the ufual remedy) have never appeared to me to afford more than a very temporary relief.

I have given the calx of zinc in large and frequent dofes, fo as to excite continual naufea, with no beneficial effects, excepting towards the latter end of one folitary inftance, when the difeafe appeared to have become purely fpafmodic. In this cafe it performed a complete cure.

The flores cardam. pratens. in fine powder, appeared to have fome effect in the fame way; but on account of the great quantity that muft be exhibited for this purpofe, they are generally inadmiffible.

In a few inftances I have cautioufly ufed volatile alk. and opium; but as this medicine never failed to aggravate the morbid difpofition of the cheft, it was quickly laid afide.

Bliftering the fternum has often relieved dyfpnœa when prefent, but never feemed to have much effect upon the cough.

Having the misfortune to lofe the firft little patient who came under my care for this difeafe laft Auguft, under the common mode of treatment, and having witneffed the good effects of the Digitalis in feveral affections of the cheft, I was induced to make trial of it.

The faturated tincture, mixed with an equal quantity of fyrup, is pleafant to the tafte, and I have hitherto experienced little or no difficulty on this account; a circumftance of fome importance where children are concerned.

Three or four cafes remain yet undecided. Of feventeen others, whofe names are now before me, thirteen have been completely cured by this medicine alone. In four others it was totally ufelefs; two of thefe had mefenteric difeafe, another was teething, and the fourth, a boy aged three years, enjoying apparent health in other refpects, never was brought under the influence of the medicine, though given to the extent of 40 drops three times a day; it was increafed no further, ther, his mother growing weary of the trouble of ufelefs exhibition. So that in three of thefe cafes the failure can hardly be afcribed to the inefficacy of the remedy, as they died when it had been given a very fhort time.

The action of the medicine has not appeared to be fo fpeedy, in children of delicate and irritable habits, as in the more vigorous and robuft.

It has never been exhibited fo as to produce perceptible ficknefs or vertigo. Beginning with a fmall dofe, it has been gradually increafed fo as to lower and leffen the frequency of the pulfe, and continued in this manner until the cough entirely ceafed.

The age of the children on whom thefe trials were made, was from eight months to four years.

In plethoric habits I have prefcribed bleeding with one or two leeches, and have begun the ufe of the tincture immediately afterwards.

Avoiding expofure to cold, particularly keeping the extremities warm, has been fedulously recommended, and has, perhaps, been a great means of preventing a recurrence of the difeafe, a circumftance very common, more efpecially at this feafon of the year; when, however, from imprudent expofure, it has recurred (as happened in one inftance) a repetition of the tincture has been attended with the beft effects.

If the above hints fhould induce practitioners to give this remedy a fair trial, and if it fhould prove fo beneficial in this hitherto almoft unconquerable complaint, as thefe inftances feem to indicate, I fhall be highly gratified.

Whilft upon this fubject, it may not be amifs to defcribe what appeared to me a fingular termination of this difeafe.

In the winter of the year 1796, I attended a child, aged five months, for the fpace of feveral weeks, and, notwithftanding the ufe of every remedy which afforded a probability of fuccefs, it continued to become gradually worfe, until its life was defpaired of. I faw it one evening, and had no expectation of feeing it alive the next day. The following morning, however, I was agreeably furprifed to find it much better; a plentiful eruption of meafles* had taken place during the night, the cough and breathing were greatly relieved, and the child had fucked very heartily. The meafles difappeared at the ufual time, the cuticle difquamated, and, from this period, every morbid fymptom vanifhed. I remain, &c.

*Hull, Dec. 26, 1800.* GEO. FIELDING, Surgeon.

---

* The meafles were exceedingly prevalent at that time all over the neighbourhood.

